# Application of Telemedicine in Gansu Province of China

**DOI:** 10.1371/journal.pone.0158026

**Published:** 2016-06-22

**Authors:** Hui Cai, Hongjing Wang, Tiankang Guo, Guoxian Bao

**Affiliations:** 1 Department of Medical Affairs, Gansu Provincial Hospital, Lanzhou, Gansu, China; 2 Gansu Provincial Hospital, Lanzhou, Gansu, China; 3 School of Management, Lanzhou University, Lanzhou, Gansu, China; University of Groningen, University Medical Center Groningen, NETHERLANDS

## Abstract

Telemedicine has become an increasingly popular option for long-distance health care and continuing education. As information and communication technology is underdeveloped in China, telemedicine develops slowly. At present, telemedicine consultation centers are situated mainly in developed cities, such as Beijing, Shanghai, and Guangzhou. In many less developed regions, such as northwest China, the conditions or related facilities are not available for the application of a better medical service. Accordingly, the aim of this paper was to introduce the construction and application of a telemedicine consultation center in Gansu Province in the northwest of China. In addition, the function of Gansu Provincial Telemedicine Consultation Center on emergency public events was introduced. As a whole, there was a great demand for telemedicine service in the local medical institutions. In the telemedicine consultation center, the telemedicine equipments and regulations were needed to be improved. The function of telemedicine service was not fully used, there was a large space to be applied and the publicity of telemedicine service was important. What is important was that telemedicine played a significance role in promoting the medical policy reform, improving the medical environment and launching the remote rescue in the emergency public events. This paper emphasizes the health care challenges of poor regions, and indicates how to share the high-quality medical service of provincial hospitals effectively and how to help residents in resource-poor environments.

## Introduction

### The Chinese telemedicine

With the advancement of information and communication technology, children and adults can receive high-quality health care from a distance through telemedicine [1]. In China, the first telemedicine consultation center was founded in The People’s Liberation Army General Hospital in 1988, where neurosurgery case discussions were carried out with doctors in Germany by satellite for the first time [2]. To date, telemedicine has a 27-year history in China. In the late 1990s, there was a shift from the theoretical exploration of telemedicine consultation to its gradual application. The National Health and Family Planning Commission (NHFPC) of the People’s Republic of China established the Chinese Jinwei Network Engineer(the National Health Information System), which promotes the development of telemedicine in China [3]. In September 1997, the China Medical Foundation established the international medical network committee, which promoted medical information and telemedicine work. In 2001, the People’s Liberation Army and NHFPC of China set up the Jun Wei II project (telemedicine network). By the end of 2001, there were about 300 net-hospitals across the whole nation, and consultation cases had risen to 1,800 [4]. In 2010, the NHFPC of China published a document about the development of remote consultations of tumor pathology and the construction of a network for quality control [5]. At present, telemedicine consultation centers are concentrated in the metropolises, such as Beijing, Shanghai, Guangzhou, and so on. However, in many areas of northwest China, the conditions or related facilities are not available for the development of telemedicine for rural residents [6].

**Fig 1:**
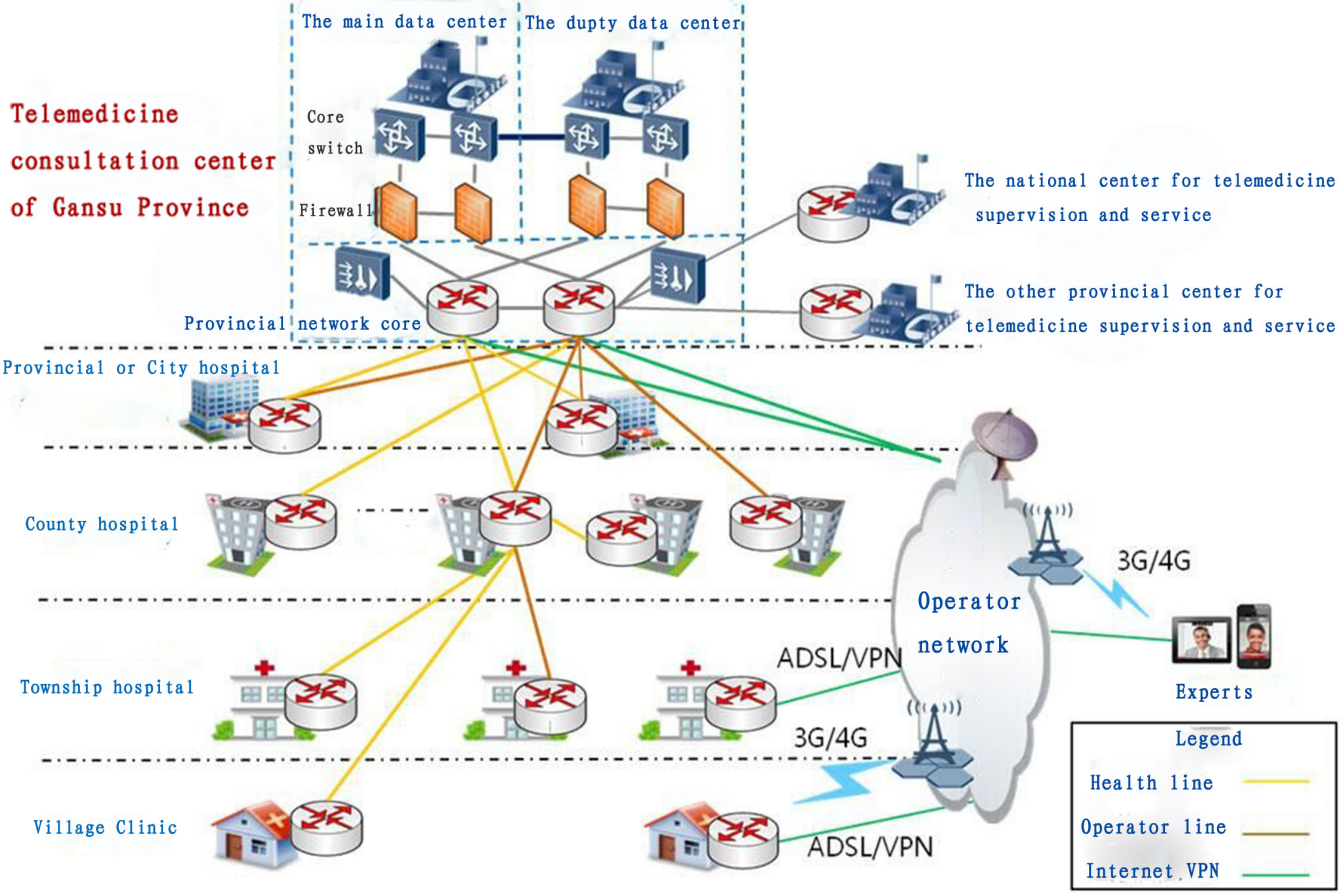
The IT infrastructure of telemedicine consultation canter of Gansu. ADSL is Asymmetric Digital Subscriber Line and VPN is Virtual Private Network.

In addtion, the infrastructure of telemedicine is important. Among the whole telemedicine system, the web service is necessary, which includes the system management and database management [6]. The structural of telemedicine are as follows. Firstly, computer networks have made it possible to share electronic medical records and to deliver medical expertise via remote consultation [7]. Secondly, the hardware is needed, such as the computers, earphones, cameras, loudspeakers, and microphones. All of the basic telemedicine can be completed using the above equipment. Other equipment is also necessary for advanced telemedicine consultation, including televisions, video conferencing equipment, and mobile phone lines. Thirdly, network security and privacy are typical problems of conducting telemedicine over the Internet. Therefore, these have to be studied well before practicing telemedicine [8]. The IT infrastructure of telemedicine could be seen in Fig 1.

The working mechanism or service process of telemedicine is crucial. The expert workers, technology, equipment, and devices must be provided by the telemedicine hospital. An agreement about the cooperation, condition, content, process, right, obligation, risk, and responsibility between the two medical institutions must also be signed before the telemedicine service can be launched. Furthermore, informed consent has to be obtained from the patients. Following this, a telemedicine invitation can be sent out. When the invited telemedicine hospital receives the signal, the decision of accepting the invitation should be made immediately. In addition, once the invitation is accepted, the telemedicine hospital needs to be well prepared. At the same time, the telemedicine hospital has to arrange for the relevant qualified doctors to provide the telemedicine service considering the laws, regulations, and diagnosis and treatment norms. Finally, a suggested treatment report has to be provided. The two medical institutions need to complete the medical record and save it well [9].

To date, the telemedicine consultation center has held consultations about imaging, pathology, intensive care, and continuing medical education. Moreover, in the near future, it will be possible to improve in some advanced fields, including electrocardiograph, endoscopy, ultrasound, surgical guidance, and so on.

### Profile of Gansu Province

Gansu Province, which is located in northwest China, covers an area of 390,000 square kilometers, with a population of 25.78 million [10]. Most areas of Gansu are plateaus and mountains, with an average altitude of above 1,000 meters, which suffer from drought. Gansu also boasts poor land resources, because it is characterized by a low percentage of utilizable land, a small proportion of cultivated land, the low capacity of the land, and a low economic level compared with other provinces in China. In 2012, the gross domestic product of Gansu was 565.02 billion yuan, accounting for 1.09% of the country’s total (51,894.21 billion yuan), and the per capita gross domestic product was 21,978 yuan, which was lower than that of the whole nation (38,420 yuan) [10]. In addition, regarding health care resources, the number of health technicians, doctors, and nurses per 1,000 people in Gansu is 4.33, 1.67, and 1.44 respectively, which is lower than the national amount (4.94, 1.94, and 1.85) [10]. It is obvious that the human resources for health care in Gansu are poor, and even the advanced medical facilities in the rural areas of Gansu are not equipped. In summary, both the income and the health services of rural residents in Gansu are lower than the average level in China.

It is difficult and expensive for many rural residents to meet the provincial hospitals’ doctors due to inconvenient traffic and a heavy burden of living. In order to improve the level of health and to relieve the burden of living of the rural residents in Gansu, the telemedicine consultation center of Gansu Province was founded in 2007, which was the first telemedicine center in northwest China and the first center to install consultation equipment for primary hospitals free of charge. The center was greatly supported by the government for utilization across the entire country. At present, the center covers all of the municipal hospitals and the county hospitals as well as the township hospitals in Gansu equipped with the telemedicine network, with the number of net-hospitals amounting to 1,487 and the number of telemedicine consultations reaching 4,000 cases per year. It is obvious that the average number of consultations is not high, and the majority of the consultations are distributed among the county level hospitals. Therefore, we can acquire that the telemedicine service system is not completely used. So, to introduce the applications of telemedicine consultation center of Gansu Province, to find the existed problems and promote using of telemedicine in poor regions is necessary.

## Materials and Methods

### Ethical consideration

Written informed consent was obtained from each participant in the study, which was approved by the ethics committee of Gansu Provincial Hospital, and was in agreement with the Helsinki Declaration of 1975, as revised in 1983. The individual in this manuscript has given written informed consent.

### Measurement and participants

To understand the proceeding situation of telemedicine consultation center of Gansu Province, find the existed problems and promote using of telemedicine in poor regions, four kinds of participants were surveyed and four questionnaires were used. The participants were all recruited from October to November 2014.

The first survey assessed the participants’ basic characteristics and the knowledge level of telemedicine, attitude towards telemedicine, and the intention to participate in telemedicine. The participants were from the primary medical institutions who were undertaking the further study in Gansu Provincial Hospital. We distributed 200 questionnaires and collected 186 valid questionnaires.

The second questionnaire was a satisfaction survey that obtained data about the participants’ basic characteristics, knowledge, and satisfaction with telemedicine. The participants were physicians who had experience of telemedicine in Gansu Provincial Hospital. One hundred questionnaires were distributed in the hospital’s telemedicine center, and 95 valid questionnaires were collected.

The third questionnaire was a survey of the administrators of the telemedicine hospital that investigated the diagnosis and treatment grading, two-way transfer of medical treatment, and the telemedicine hospital’s publicity. Thirty-one administrators were surveyed.

The last questionnaire was a satisfaction survey of the patients who had experienced the telemedicine service. The questionnaire assessed the level of knowledge of telemedicine, convenience of telemedicine, satisfaction with the treatment outcome using telemedicine and the medical fees. The patients were recruited from the directional supported county (a Chinese directional support project that the provincial institutions should assist a local county) by the simple random sampling method. A total of 360 patients were interviewed and all questionnaires were valid.

In addition, the function of Gansu Provincial Telemedicine Consultation Center in the emergency public events was introduced.

### Analysis

Descriptive statistics were used to describe the participants’ basic characteristics, knowledge of telemedicine, attitude towards telemedicine, intention of participating in telemedicine, and satisfaction with telemedicine.

## Results

### Basic characteristics

The total number of investigated primary medical workers was 186. Of the workers, 102 (54.8%) were male, 84 (45.2%) were female, 82 (44.1%) were residents, 73 (39.2%) were assistants, 15 (8.1%) were deputy chief physicians, seven (3.8%) were chief physicians, and nine (4.8%) had no title. Furthermore, 95 physicians from Gansu Provincial Hospital were investigated. Of these, 62 (65.3%) were male, 33 (34.7%) were female, 52 (54.7%) were deputy chief physicians, 43 (45.3%) were chief physicians, and there were no residents or assistants.

Of the investigated administrators, 21 (67.7%) were male and 10 (32.3%) were female. The mean age was 45 years with a *SD* of 5.3. Every administrator was a medicine major. Of the surveyed patients, 205 (56.9%) were male and 155 (43.1%) were female. The mean age was 53 years with a *SD* of 6.5. All of the diseases that were consulted about using telemedicine were distributed among internal medicine, surgery, gynecology, and pediatrics.

### The survey of the intention of the primary medical workers

As shown in Table 1, 31(16.7%) primary medical workers knew the knowledge of telemedicine very well. Sixty-two (33.3%) thought that the unbalanced health resources could be resolved by telemedicine usefully, while 32(17.2%) thought that it was useless. Among the workers, 181 (97.3%) expressed that if there was an opportunity and they would like to participate in telemedicine.

**Table 1:** The Survey of the Intention of Medical Workers from Primary Medical Institutions (*N* = 186).

Items	*n*	%
**Do you know about telemedicine?**		
No	8	4.3
A little	65	34.9
Yes	82	44.1
Very well	31	16.7
**Could unbalanced health resources be resolved by telemedicine?**		
Not sure	24	12.9
Useless	32	17.2
Somewhat useful	68	36.6
Very useful	62	33.3
**Do you want to participate in telemedicine?**		
No	5	2.7
Yes	181	97.3

### The survey of physicians’ satisfaction with the experience of using telemedicine

As shown in Table 2, of the physicians who completed the survey about the experience of using telemedicine in Gansu Provincial Hospital, 65 (68.4%) and 59 (62.11%) physicians had participated in telemedicine consultation and treatment mainly. All of the 95 (100.0%) physicians had participated in the unified training about telemedicine previously. Twenty-one (22.1%) physicians thought that the operation of the telemedicine interface was complicated. Forty (42.1%) physicians were satisfied with the telemedicine’s image quality. Thirty-two (33.7%) physicians were satisfied with the transmission speed of the telemedicine. Seventy-eight (82.1%) physicians wanted to participate in telemedicine in the future, while 17 (17.9%) physicians did not. Of the 17 physicians who did not want to participate in telemedicine in the future, the main reasons were that they thought that the laws and regulations about telemedicine were not perfect and the transportation was convenient. Overall, the lower percentage of physicians (28.4%) was satisfied with the telemedicine of Gansu Provincial Hospital, and about fifty percentages of physicians had conservative attitude.

**Table 2:** The Survey of Physicians’ Satisfaction with the Experience of Telemedicine in Gansu Provincial Hospital (*N* = 95).

Items	*n*	%
**In which aspects of telemedicine did you participate?**		
Education	23	24.2
Consulting	36	37.9
Consultation	65	68.4
Conference	27	28.4
Treatment	59	62.1
Monitoring	41	43.2
**Did you participate in the unified training about telemedicine?**		
No	0	0.0
Yes	95	100.0
**What about the operation of the telemedicine interface?**		
Easy	36	37.9
Neutral	38	40.0
Complicated	21	22.1
**What about the image quality of the telemedicine?**		
Unsatisfied	21	21.1
Neutral	34	35.8
Satisfied	40	42.1
**What about the transmission speed of the telemedicine?**		
Unsatisfied	24	25.3
Neutral	39	41.0
Satisfied	32	33.7
**Do you want to participate in telemedicine in the future?**		
No	17	17.9
Yes	78	82.1
**Why do you not want to participate in telemedicine in the future?**		
It is not reliable	0	0.0
Laws and regulations are not perfect	5	29.4
The management system of the hospital is not complete	4	23.5
Being used to the traditional medical mode	3	17.7
The transportation is convenient	5	29.4
**Were you satisfied with the telemedicine of Gansu Provincial Hospital?**		
Unsatisfied	16	16.8
Neutral	52	54.8
Satisfied	27	28.4

### The survey of the administrators of the telemedicine hospital

To understand the effect of telemedicine on medical system reform, the administrators of the telemedicine hospital were surveyed. As shown in Table 3, 29 (93.5%) and 28 (90.3%) administrators thought that telemedicine was useful for diagnosis and treatment grading, and for the two-way transfer of medical treatment, respectively. All of the administrators indicated that telemedicine was helpful for the telemedicine hospital’s publicity.

**Table 3:** The Survey of the Administrators of the Telemedicine Hospital (*N* = 31).

Items	*n*	%
**Was telemedicine useful for diagnosis and treatment grading?**		
No	2	6.5
Yes	29	93.5
**Was telemedicine useful for the two-way transfer of medical treatment?**		
No	3	9.7
Yes	28	90.3
**Was telemedicine helpful for the telemedicine hospital’s publicity?**		
No	0	0.0
Yes	31	100.0

### The survey of the satisfaction of patients who had experienced the telemedicine service

As shown in Table 4, 204 (56.67%) patients knew the telemedicine service well, and 156 (43.3%) patients knew it a little. Of the patients, 382 (78.3%) thought that seeing the doctor via telemedicine was convenient, but 78 (21.7%) patients thought that telemedicine was not convenient. Two hundred (55.5%) patients were satisfied with the treatment result by telemedicine, 123 (34.2%) patients were neutral and 37 (10.3%) patients were unsatisfied with the treatment result. Of the patients, 277 (76.9%) were satisfied with the medical fees, 64 (17.8%) patients were neutral and 19 (5.3%) patients were unsatisfied.

**Table 4:** The Survey of the Satisfaction of Patients Who Had Experienced the Telemedicine Service (*N* = 360).

Items	*n*	%
**Did you know the telemedicine service?**		
No	0	0.0
A little	156	43.3
Yes	204	56.7
**Was telemedicine convenient?**		
No	78	21.7
Yes	382	78.3
**What about the treatment result by telemedicine?**		
Unsatisfied	37	10.3
Neutral	123	34.2
Satisfied	200	55.5
**What about the medical fees?**		
Unsatisfied	19	5.3
Neutral	64	17.8
Satisfied	277	76.9

### The function of Telemedicine Consultation in the emergency public events

In recent years, Gansu Provincial Telemedicine Consultation Center has functioned sufficiently when there have been emergency public events, such as natural disasters (e.g., the 2013 Dingxi earthquake, 2010 Zhouqu mudslide, and 2008 Wenchuan earthquake), public health events (e.g., influenza A [H1N1] and the melamine milk powder incident), accident disasters (e.g., the 2011 Qingyang bus accidents and 2010 Longnan crash), and so on. Table 5 shows several cases in sudden public events that were consulted about using the telemedicine consultation center.

**Table 5:** The Cases Consulted in Several Sudden Public Events.

The sudden public events	Date	Cases
**The natural disasters**		
Dingxi earthquake	July 22, 2013	54
Zhouqu earthquake	Aug 7, 2010	10
Wenchuan earthquake	May 12, 2008	12
**The public health events**		
Influenza A (H1N1)	2009	15
The melamine milk powder incident	2008	13
**The accident disasters**		
Qingyang bus accidents	Nov 16, 2011	48
Longnan crash	Oct 26, 2010	6

The 2013 Dingxi earthquake is a typical example. On July 22, 2013, the 6.6 magnitude earthquake struck Dingxi City of Gansu Province. The telemedicine consultation center opened the emergency rescue platform immediately and launched online treatment services free of charge. In the disaster-stricken areas, some patients who suffered severe injuries, and who might not have been diagnosed accurately by the local physicians, were diagnosed by the experts from Gansu Provincial Hospital using telemedicine, and the decision was made whether the patients could receive symptomatic treatment. Subsequently, many patients who could not be treated were transferred to the provincial hospital. Gansu Provincial Hospital received 23 patients from the disaster-stricken area. A total of 54 injured people in the disaster-stricken areas were consulted. Besides being used for diagnosis and treatment, the telemedicine system could assist the government in announcing an outbreak at the earliest time and offering training for the medical workers in disaster areas.

## Discussion

In Gansu, the per capita annual cash living expenditure of rural residents is 3,689.03 yuan, which is lower than the standard level of the whole nation (5,414.47 yuan) [10]. For the per capita annual cash living expenditure of rural residents, health care and medical services, transportation and communication account for 10.79% and 11.82%, respectively [10]. And for the rural residents of Gansu, there is a great lack of health awareness, and the education level is relatively low. Of the population aged 6 years and above, 8.82% have no educational experience at all. This percentage is quite high when compared with other areas in China [6], and the percentage of rural residents who have a college degree or experience of higher education amounts to only 8.90% [10]. Many patients concentrate in provincial hospital, while there are no or less patients in primary medical institutions. Also primary medical institutions lack excellent medical talents, and the medical service ability is low. As result, there is a phenomenon that residents see the doctor difficultly and the medical fee is expensive. As such, it is necessary to improve the medical environment, promote the medical information construction, improve the treatment capacity and collaboration service levels of local health care team and improve public health consciousness and self health management ability.

Of all the telemedicine services that had been launched, only the consultation and treatment through telemedicine were mainly applied. Therefore, the other telemedicine services should be extended and the good publicity is needed about the telemedicine business. Telemedicine training, education about health, and awareness of diseases is crucial and urgent for the rural residents. The local physicians could obtain new and advanced health knowledge and technology, and academic exchange, free training, and operation guidance could be launched. In the telemedicine hospital, every doctor who joined in the telemedicine had been trained in the standard level required to meet the service regulations and protocols. In addition, the operation interface, image quality, and transmission speed of the telemedicine system needs to be improved. Most of the doctors wanted to participate in the telemedicine continuously, but about 18% of the physicians did not want to participate in telemedicine in the future. The main reason given was that the laws and regulations of telemedicine were not perfect. Nowadays, patients from rural areas can travel to the provincial hospital using a convenient transportation system. However, in Gansu, there are still many people who live in remote mountain areas.

Moreover, launching telemedicine could be effective in strengthening the communication between the doctors of provincial hospitals and the doctors of local hospitals. It also could promote the development of medical reform in China, such as for the two-way transfer of medical treatment, and the diagnosis and treatment grading [8]. Telemedicine could improve the stay of patients with minor illnesses in the local hospitals, and only patients with major illnesses might be transported to the provincial hospitals. It is promising that telemedicine could play an important role in balancing the current medical resources. In general, the unified goal of telemedicine and the medical reform policy was to improve the medical environment of China.

According to the results, a part of the patients thought that the telemedicine was not convenient. And they were used to seeing doctors face-to-face in the traditional way, which they thought it was a trustworthy way. However, the patients with experience of telemedicine were satisfied with the telemedicine’s advantages, for example, the medical fees [11]. And also launching telemedicine could reduce the number of repeated medical tests and lower the costs, such as transportation fees and the cost to the accompanying persons, etc [12]. The above contents showed that some patients had lower knowledge level for telemedicine, they didn’t know the benefits of telemedicine, and so it was critical to let more patients enjoy telemedicine service.

In 2013, the NHFPC of China renewed the program for extending targeted aid in urban and rural hospitals. In this project, in order to launch the consultation of complicated and serious diseases as a priority, it was also necessary to develop the pathological diagnosis and continuing education using the telemedicine consultation system [13]. This policy greatly promoted the development of telemedicine consultation in Gansu.

In a word, we found that the knowledge level for telemedicine of rural areas was low, but there was a great demand for telemedicine service in the local medical institutions. In the telemedicine consultation center, the telemedicine equipments and regulations were needed to be improved. Therefore, it is suggested that the government increases financial input into the telemedicine infrastructure for rural areas and formulates more medical policies that could enhance the effective use of the high-quality medical service in order to benefit rural residents [6]. It also concluded that the telemedicine service was not fully used, there was a large space to be applied and the publicity of telemedicine service was important. What is important was that telemedicine played a significance role in promoting the medical policy reform, improving the medical environment and launching the remote rescue in the emergency public events.

Since Gansu Provincial Telemedicine Consultation Center was established in 2007, it has had regular communication with other domestic telemedicine consultation centers, such as the telemedicine consultation center of Huaxi medical college and Xiangya medical college. In 2009, the experts from Gansu Provincial Telemedicine Consultation Center communicated with specialists in Oklahoma State University via the telemedicine system. Moreover, in October 2010, telemedicine consultation was launched between Gansu Provincial Hospital and Taiwan Provincial Taipei Hospital. The two consultations mentioned above were a big leap forward for Gansu Provincial Telemedicine Consultation Center. However, as telemedicine consultation is limited to only being between medical institutions, it could be possible in the future to introduce the use of telemedicine into the family for their health care [14].

## References

[1] Nesbitt TS. Meeting the health care needs of California’s children: The role of telemedicine. 2007. Available: http://www.aucd.org/docs/resources/Childrensdoc.pdf.

[2] Zhang C, Wang GX, Xi HB. The development and status of the telemedicine at home and abroad. Zhonghua Yi Xue Za Zhi. 2010; 90: 1726–1728. Chinese.

[3] Gao YJ. The process of Jinwei network engineering. Chinese Journal of Health Statistics. 1998; 5: 5–6. Chinese.

[4] Jin GQ, Zhang KJ, Cui DX. The situations of telemedicine and the analysis of the future application. Medical Journal of National Defending Forces in Southwest China. 2001; 11: 211–213. Chinese.

[5] The National Health and Family Planning Commission of the People’s Republic of China. Developing the pathological remote consultation of tumor and the construction of network in quality control. Available: http://www.nhfpc.gov.cn/yzygj/s3590/201009/a46d4f454b58438a86ed26a4a89413ab.shtml.

[6] Hsieh RKC, Hjelm MN, Lee JCK, Aldis JW. Telemedicine in China. Int J Med Inform. 2001; 61: 139–146. 11311668 10.1016/s1386-5056(01)00136-8

[7] Chen Z, Yu XM, Feng D. A telemedicine system over the internet. Visual Information Processing—VIP. 2000.

[8] Zhao J, Cai YL, Sun DX, Zhai YK. Discussing the status of the development of telemedicine and its trend. The Chinese Health Service Management. 2014; 10: 739–740. Chinese.

[9] Tsirintani M.. Strategic procedures and revisions for implementing telemedicine and telecare in Greece. Applied Clinical Informatics. 2012; 3: 14–23. doi: 10.4338/ACI-2011-08-R-0048 23616897 10.4338/ACI-2011-08-R-0048 PMC3613008

[10] National Bureau of Statistics of China. China Statistical Yearbook of 2013. China Statistics Press Available: http://www.stats.gov.cn/tjsj/ndsj/2013/indexch.htm.

[11] Kamsu-Foguem B, Foguem C. Could telemedicine enhance traditional medicine practices? European Research in Telemedicine. 2014; 3: 117–123.

[12] Li X, Valentino DJ, George J. A World Wide Web telemedicine System. Proceedings of SPIE Medical Imaging. PACS: Design and Evaluation. 1996; 2711: 427–439.

[13] The National Health and Family Planning Commission of the People’s Republic of China. Deepening the targeted aid in the urban and rural hospitals. Available: http://www.moh.gov.cn/yzygj/s3594r/201309/b73be8538ce24765ab167ec1fa621ddd.shtml.

[14] Bashshur R, Krupinski E, Grigsby J. The taxonomy of telemedicine. Telemedicine Journal and e-Health. 2011; 17: 484–494. doi: 10.1089/tmj.2011.0103 21718114 10.1089/tmj.2011.0103

